# Pressurized intraperitoneal aerosol chemotherapy (PIPAC) as a neoadjuvant therapy before cytoreductive surgery and hyperthermic intraperitoneal chemotherapy

**DOI:** 10.1186/s12957-016-1008-0

**Published:** 2016-09-27

**Authors:** Ramy Girshally, Cedric Demtröder, Nurettin Albayrak, Jürgen Zieren, Clemens Tempfer, Marc A. Reymond

**Affiliations:** 1 Therapy center for peritoneal diseases, Elisabethengruppe, Herne, Germany; 2 Ruhr-University Bochum, Bochum, Germany; 3 Department of Surgery, University of Tübingen, Hoppe-Seyler Str. 3, T2076 Tübingen, Germany

**Keywords:** Peritoneal metastasis, Intraperitoneal chemotherapy, Cytoreductive surgery, Hyperthermic intraperitoneal chemotherapy (HIPEC), Pressurized intraperitoneal aerosol chemotherapy (PIPAC), Cisplatin, Doxorubicin

## Abstract

**Background:**

Pressurized intraperitoneal aerosol chemotherapy (PIPAC) is a novel drug delivery system able to induce regression of peritoneal metastasis (PM) in the salvage situation. The aim of this study was to determine the clinical characteristics, tumor histology, and extent of disease of the patients having undergone cytoreductive surgery (CRS) and hyperthermic intraperitoneal chemotherapy (HIPEC) after “neoadjuvant” PIPAC.

**Methods:**

This study was performed at a single institution, tertiary center. In a prospective registry, retrospective analysis was done. PIPAC indication was restricted to patients in the salvage situation who were not eligible for cytoreductive surgery (CRS) and hyperthermic intraperitoneal chemotherapy (HIPEC).

**Results:**

Nine-hundred sixty-one PIPAC sessions were successfully performed in 406 patients: 21 patients (5.2 %) were scheduled for CRS and HIPEC. Twelve of these patients had a low PCI (mean 5.8 ± 5.6). The remaining nine patients showed an advanced peritoneal disease (mean PCI 14.3 ± 5.3) at initial laparoscopy. After repeated PIPAC (mean number of cycles 3.5 ± 0.9), radiological tumor regression was observed in 7/9 patients and major histological regression was observed in 8/9 patients, so that secondary CRS and HIPEC became possible.

**Conclusions:**

PIPAC might be used as a neoadjuvant therapy before CRS and HIPEC in order to improve the outcome of CRS and HIPEC, to select patients with chemosensitive, biologically favorable tumors, to extent the indications of CRS and HIPEC in the presence of diffuse small bowel involvement, and to reduce the extent of cytoreductive surgery.

## Background

Peritoneal metastasis is a common and dismal evolution of several gastrointestinal and gynecological tumors, including gastric, ovarian, colorectal, hepatobiliary, pancreatic, uterine, urological, and other cancers [1]. The therapy of peritoneal metastasis is largely palliative; with the aim of prolonging life and preserving its quality. Most patients receive platin-based, combination systemic chemotherapy [2]. In spite of this guideline-recommended therapy, they die within months after diagnosis of peritoneal dissemination [3].

Almost 70 years ago, intraperitoneal chemotherapy has been discovered as an alternative therapeutic option in peritoneal metastasis [4]. In the meantime, a significant pharmacological advantage of intraperitoneal chemotherapy was documented in the preclinical model, and numerous clinical studies have delivered promising clinical results (reviewed in [5]). In the last 30 years, cytoreductive surgery (CRS) combined with hyperthermic intraperitoneal chemotherapy (HIPEC) has been increasingly used. On the basis of long-term survivors, some authors see a curative role for this combined therapy [6]. However, the level of evidence of CRS and HIPEC is still relatively low, and the complication rate remains significant so that this therapy is not accepted by all oncologists [7].

In spite of the above controversies, there is a broad agreement that CRS and HIPEC should only be offered to highly selected patients, taking into consideration the tumor type, the extent of disease, and the general condition of the patient [8]. In particular, diffuse invasion of the small bowel represent a contraindication for CRS and HIPEC because of the dilemma between complete cytoreduction and extensive resection of the small bowel—which is not compatible with life [9]. Thus, there is an urgent need for novel therapies for the majority of peritoneal metastasis patients not eligible for CRS and HIPEC.

Pressurized intraperitoneal aerosol chemotherapy (PIPAC) is an innovative approach delivering chemotherapy into the peritoneal cavity as a pressurized normothermic aerosol [10]. Early studies in patients with peritoneal metastases secondary to ovarian [11] or other abdominal cancers [12, 13] have shown some efficacy and good tolerability, in particular PIPAC does not induce significant neither liver or renal toxicity [14] nor gastrointestinal symptoms [15]. In summer 2015, an independent technology assessment concluded that “As an experimental technique, PIPAC has been used in patients who are quite ill and have already failed multiple treatment regimes, but it may not be limited to that group of patients in the future. PIPAC may have significant advantages over existing chemotherapy techniques, which are painful and disabling, and associated with long length of stay and a high risk of adverse events” [16]. Several clinical trials on PIPAC in various indications are ongoing [17–22]. However, it is difficult to assess the safety and effectiveness of PIPAC since no comparative studies have been published so far.

Our institution is pioneering the potential fields of the application of PIPAC, including defining indications and contraindications, chances and risks, as well as success and failures of this therapy. We have observed repeatedly that some patients who were primarily not eligible for CRS and HIPEC, most often because of small bowel involvement, could be treated after repeated PIPAC application with CRS and HIPEC.

The general aim of this study was to determine the clinical characteristics, tumor histology, and extent of disease of the patients having undergone CRS and HIPEC after “neoadjuvant” PIPAC. We also determined the survival of these patients having received this sequential therapy, in association or not with systemic palliative chemotherapy.

## Methods

### Study design

Retrospective analysis of data obtained within the framework of a prospective registry (observational study). No blinding was applied.

### Setting

This study was performed at a university hospital, tertiary care center, certified interdisciplinary center for therapy of peritoneal disease.

#### Patient selection

Before therapy, each patient was presented in the interdisciplinary tumor board and the indication for therapy was decided on a case-by-case, individual basis. Eligible patients were treated with cytoreductive surgery (CRS) and hyperthermic intraperitoneal chemotherapy (HIPEC). No inclusion and exclusion criteria were pre-defined. All consecutive patients admitted for PIPAC and/or HIPEC in our institution between February 3, 2010, and March 16, 2016, were included.

### Technique of PIPAC

The technique of PIPAC has been described elsewhere [23]. Shortly, after insufflation of a 12 mmHg CO_2_ pneumoperitoneum with open access or with Veres needle, two balloon safety trocars (5 and 12 mm, Applied Medical, Düsseldorf, Germany) were inserted into the abdominal wall. The extent of peritoneal carcinomatosis (PCI score) was determined based on lesion size and distribution [24]. Peritoneal biopsies were taken in all four quadrants for histological examination, and a local partial peritonectomy of several square centimeters was performed routinely to improve accuracy of anatomopathology. A 9-mm aerosolizer (Capnopen®, Capnomed, Villingendorf, Germany) was connected to an intravenous high-pressure injector (Arterion Mark 7®, Medrad, Bayer, Germany) and inserted into the abdomen through an access port. Following safety measures were taken to exclude any exposure of the operating team [25]. First, tightness of the abdomen was documented via a zero flow of CO_2_. Second, the procedure was performed in an operating room equipped with laminar air flow. Third, chemotherapy injection was remote-controlled and nobody remained in the operating room during the application. For patients with ovarian [11], gastric [12], and hepatobiliary-pancreatic [26] cancers, a pressurized aerosol containing doxorubicin at a dose of 1.5 mg/m^2^ body surface in a 50 ml NaCl 0.9 % solution followed by cisplatin at a dose of 7.5 mg/m^2^ body surface in a 150 ml NaCl 0.9 % solution was applied via aerosolizer and injector. For colorectal and appendiceal cancer patients, oxaliplatin at a dose of 92 mg/m^2^ was applied instead of cisplatin and doxorubicin, as described elsewhere [13]. Flow rate was 30 ml/min and maximal upstream pressure was 200 psi (13.8 bar). The therapeutic capnoperitoneum was then maintained for 30 min at 37 °C. Then, the chemotherapy aerosol was exsufflated via a closed line over two sequential microparticle filters into the airwaste system of the hospital. Finally, trocars were retracted and laparoscopy was ended. No drainage of the abdomen was applied. If possible, the PIPAC procedure was repeated after 6 weeks.

### Technique of CRS

Cytoreductive surgery was performed as described elsewhere [27], with the aim of complete cytoreduction (CC-0). When necessary, radical cytoreduction was associated with multivisceral resection, including resection of the diseased peritoneum in all the four abdominal quadrants, in the pelvis and a total omentectomy. Normal appearing peritoneum was not removed. Visceral resections included right colectomy, sigmoid colon resection, splenectomy, distal pancreatectomy, atypical liver resection, partial gastrectomy, and partial diaphragmatic resections. However, the aim was to preserve as many organs as possible. In particular, tumor nodules located on the peritoneal surface of the small and large bowel were removed without organ resection when no infiltration was present.

### Technique of HIPEC

Following CRS, HIPEC was performed using the closed technique for 60 min at a temperature of 41–43 °C using extracorporal circulation of liquid solutions. HIPEC was initiated after bowel reconstruction and abdominal closure. The drugs applied were oxaliplatin 360 mg/m^2^ body surface for appendiceal and colorectal cancer cases or a combination of cisplatin 75 mg/m^2^ body surface and doxorubicin 15 mg/m^2^ body surface in the other cancer types. In colorectal cases, 5-fluorouracil was infused immediately after the procedure to enhance the effects of hyperthermic intraperitoneal oxaliplatin, as described elsewhere [28].

#### Completeness of cytoreduction

Completeness of cytoreduction score (CC score) was determined according to Sugarbaker [29]: CC-0: no residual disease; CC-1: residual disease <2.5 mm; CC-2: residual disease >2.5 mm.

#### Karnofsky index (KI)

The Karnofsky index [30] was determined at the time point of hospital admission in all patients and was used for estimating prognosis and defining therapeutic goals. The index scale ranges from 0 (death) to 100 % (no restriction).

#### Follow-up

Patients were followed up until March 21st, 2016, or until death. This short follow-up is explained by the prospective nature of the prospective PIPAC registry, which is updated daily.

#### Radiological criteria of tumor regression

Repeated CT scans were performed at 3 to 6 months interval, and radiological response was assessed according to RECIST 1.1 criteria [31].

#### Histological criteria of tumor regression

Macroscopically, it was possible to determine a general pattern of tumor regression (glassy tumor nodes with hard consistence, progressive scarring, disparition of tumor neovessels, and vanishing of ascites). However, it is not possible to distinguish as a surgeon between scar tissue and residual tumor node. For such purpose, histological analysis is mandatory. The histopathological response (regression grading) was assessed by an independent pathologist as follows: tumor regression grading (TRG) 0 indicated a tumor without regression; TRG1 indicated a dominant tumor mass with obvious fibrosis and/or vasculopathy; TRG2 indicated dominantly fibrotic changes with few tumor cells or groups that were easy to identify; TRG3 described only very few tumor cells that were difficult to locate in the fibrotic tissue with/without mucous substance; and TRG4 indicated that only a fibrotic mass without tumor cells was present, i.e., total regression or response [32]. The pathologist was blinded to the macroscopic and clinical outcomes but was able to compare the biopsies to the previous PIPACs. At the time patients were operated on, the Peritoneal Regression Grading Score (PRGS) had not yet been proposed [33].

#### Statistical analysis

This is a retrospective case series. No size sample was defined a priori, since this is an exploratory study aimed at generating pilot data. Statistics were performed using the SPSS version 22.0 software. Descriptive statistics included mean and standard deviation, median, percentiles, and confidence interval. Survival statistics were computed and plotted according to Kaplan-Meyer. For survival statistics, a log-rank test (with or without linear trend depending on the question) was calculated. *P* < 0.05 was considered significant.

## Results

**Fig. 1:**
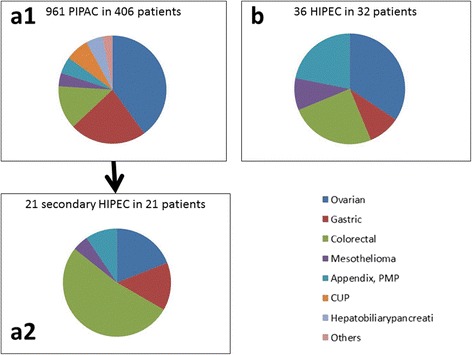
Indications for PIPAC (**a1**), for CRS and HIPEC (**b**) for CRS and HIPEC after “neoadjuvant” PIPAC (**a2**). In our institution, primary indications for PIPAC and HIPEC are relatively similar with a majority of ovarian cancers. However, indication for PIPAC was 12× more frequent than indication for CRS and HIPEC. Secondary CRS and HIPEC were performed in 5.1 % of PIPAC patients with a majority of colorectal cancer patients. PIPAC might allow secondary CRS and HIPEC in selected patients with colorectal cancer who were not eligible primarily for such procedure

During the period of investigation, 961 PIPAC sessions were successfully performed in 406 patients, in the mean of 2.3 PIPAC cycles per patient. Out of these 406 patients treated with PIPAC, 21 (5.2 %) were scheduled for CRS and HIPEC during the later course of therapy. During the same period of time in our institution, 36 other CRS and HIPEC were performed in 32 patients, independently from any PIPAC indication or therapy. CRS and HIPEC were repeated in four patients. The characteristics of all 53 patients treated with CRS and HIPEC are summarized in Table 1. A flow diagram is presented in Fig. 1, which précises the organ of origin of peritoneal metastasis

**Table 1:** Clinical characteristics of 53 consecutive patients treated with CRS and HIPEC between February 2010 and March 2016 at our institution

Patients	All	Primary CRS and HIPEC	Secondary CRS and HIPEC (after PIPAC)
Number of patients	53	32	21
Age (years)	55.8 ± 9.5	55.3 ± 9.6	56.3 ± 9.5
Sex (M:F)	20:33	8:24	12:9
Karnofsky index (%)	86.0 ± 10.1	85.9 ± 11.0	92.1 ± 5.6
Organ			
-Ovarian	15 (28 %)	11 (35 %)	4 (19 %)
-Colorectal/appendix	19 (36 %)	8 (25 %)	11 (52 %)
-PMP	9 (17 %)	7 (22 %)	2 (10 %)
-Gastric	6 (11 %)	3 (9 %)	3 (14 %)
-Mesothelioma	4 (8 %)	3 (9 %)	1 (5 %)
Peritoneal Carcinomatosis index (initial PCI)	12 ± 8	13.3 ± 9.0	11.5 ± 6.4
Ascites (ml)	147 ± 466	0 ± 0	198 ± 536

### Therapy and results

When examining in detail the clinical files of all 53 patients treated with CRS and HIPEC, we found that 21 patients were treated after neoadjuvant PIPAC in this palliative situation. Out of these 21 patients, 12 patients were scheduled for CRS and HIPEC after the first PIPAC because the intraoperative finding showed a low PCI (mean 5.8 ± 5.6). Clearly, these cases had not been evaluated previously for possible therapy with CRS and HIPEC. In these 12 patients, CRS and HIPEC were indeed possible independently of previous PIPAC, even though a single PIPAC cycle was performed in these patients.

### PIPAC as a neoadjuvant therapy

**Fig. 2:**
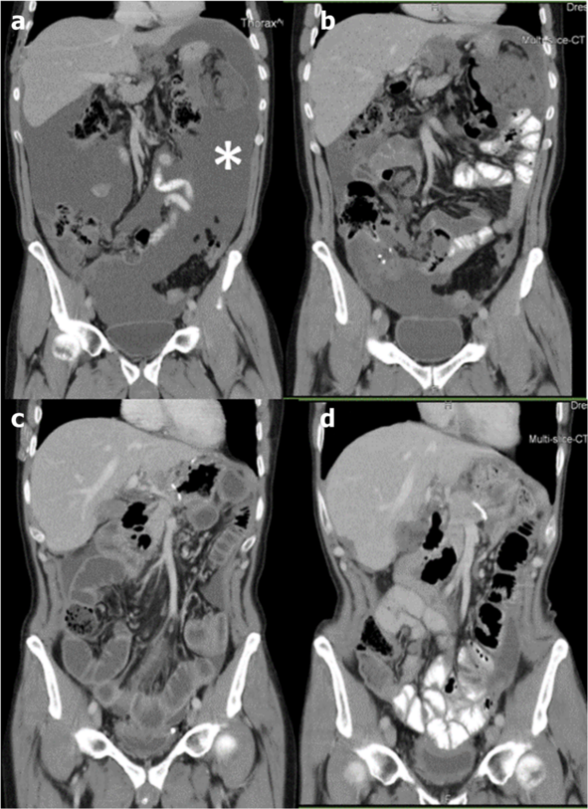
Contrast-enhanced CT scans of a 57-year-old male patient with peritoneal metastasis of an appendiceal cancer. **a** Image after 12 cycles of combination palliative chemotherapy with FOLFOX4 and 6 cycles of FOLFIRI showing active disease with massive ascites (*asterisk*). **b** Evolution after 5 cycles of PIPAC with low-dose cisplatin and doxorubicin showing partial tumor response according to RECIST 1.1 criteria, in particular ascites control. **c** Postoperative image after cytoreductive surgery and hyperthermic intraperitoneal chemotherapy (HIPEC). **d** CT scan 6 months after CRS and HIPEC showing beginning recurrence (minimal ascites and tumor node on the lateral liver surface). The patient survived 46 months after diagnosis, 25 months after first PIPAC, and 18 months after CRS and HIPEC

The remaining nine patients showed at initial laparoscopy such an extent of peritoneal disease that they were no candidates for primary CRS and HIPEC. At first PIPAC, PCI was relatively high (mean 14.3 ± 5.3) and complete cytoreduction (CC-0) did not appear feasible, in particular because of diffuse small bowel involvement. Six of these nine patients had colorectal or appendiceal cancer, one ovarian cancer, one malignant peritoneal mesothelioma, and one advanced pseudomyxoma peritonei. In these nine patients, an objective tumor regression was observed after repeated PIPAC (mean number of cycles 3.5 ± 0.9), so that secondary CRS and HIPEC were possible. The procedures are summarized in Table 2. Objective radiological tumor regression according to RECIST 1.1 criteria was observed in seven out of nine patients. An example of such regression is shown in Fig. 2. Objective major histological regression was observed in eight out of nine patients, minor regression in the last patient.

**Table 2:** CRS and HIPEC procedures performed in 53 patients with (*n* = 21) or without (*n* = 32) “neoajuvant” PIPAC

Patients	All (*n* = 53)	Primary CRS and HIPEC (*n* = 32)	Secondary CRS and HIPEC (after PIPAC) (*n* = 21)
Number of CRS and HIPEC -Single procedure -Repeated HIPEC	57 53 4	36 32 4	21 21 0
Completeness of cytoreduction (CC) score -CC-0 -CC-1 -CC-2	39 (74 %) 12 (22 %) 2 (4 %)	22 (69 %) 9 (28 %) 1 (3 %)	17 (81 %) 3 (14 %) 1 (5 %)
Number of PIPAC before HIPEC 1 2 3 4 5	12 0 4 4 1	N/A N/A N/A N/A N/A	12 0 4 4 1
Downstaging^a^ -Histology -Radiology	N/A N/A	N/A N/A	8/9 7/9

*N*/*A* non available

^a^Out of nine patients with ≥2 PIPAC

### Survival

**Fig. 3:**
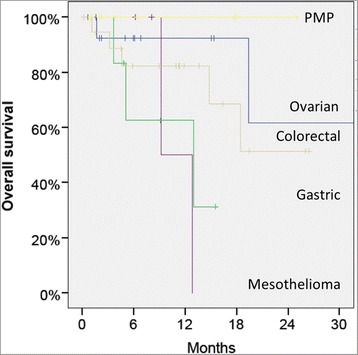
Overall survival of 53 patients treated with CRS and HIPEC, grouped according to the organ of origin. Best survival is observed in pseudomyxoma peritonei patients (*n* = 9), followed by ovarian (*n* = 15) and colorectal (*n* = 19) cancer patients. In this retrospective cohort, selected patients with gastric cancer (*n* = 6) and malignant mesothelioma (*n* = 4) have the worst prognosis

Figure 3 shows the overall probability of survival of 53 patients treated with CRS and HIPEC, grouped according to the organ of origin. Best survival was observed in pseudomyxoma peritonei patients (*n* = 9), followed by ovarian (*n* = 15), and colorectal (*n* = 19) cancer patients. In this retrospective cohort, selected patients with gastric cancer (*n* = 6) and malignant mesothelioma (*n* = 4) had the worst prognosis. One patient died in the hospital on postoperative day 33.

**Fig. 4:**
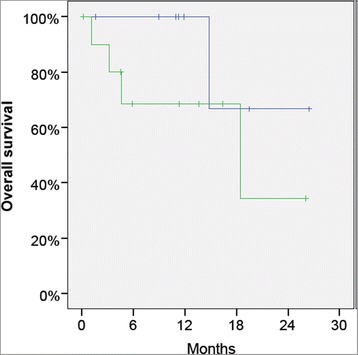
Overall survival of 19 patients with peritoneal metastasis treated with CRS and HIPEC, with (*green curve*) or without (*blue curve*) “neoadjuvant” PIPAC. As expected, patients primarily not eligible for CRS and HIPEC and treated with neoadjuvant PIPAC seem to have a worse prognosis than the other patients. However, this difference does not reach statistical significance in this small cohort of patients

We also examined the survival probability in the patients with peritoneal metastasis of colorectal origin (*n* = 19) depending on a neoadjuvant therapy with PIPAC before CRS and HIPEC. The survival curve shows that the patients with extensive disease, pre-treated with PIPAC, had a worse prognosis than those treated primarily with CRS and HIPEC in the presence of limited disease (Fig. 4). It has to be noted that these patients are not directly comparable since the patients treated with neoadjuvant PIPAC were primarily no candidates for CRS and HIPEC because of the extension of peritoneal disease. Interestingly, this difference in survival probability does not reach statistical significance, but this should be interpreted with caution because of the small number of patients and of the retrospective nature of this analysis.

We performed an exploratory multivariate analysis in order to identify potential prognostic factors for survival in this cohort of 53 patients. Three variables were identified that approached statistical significance: therapy with neoadjuvant PIPAC (*p* = 0.08), PCI score (*p* = 0.19), and organ of origin (*p* = 0.27).

## Discussion

This exploratory analysis is the first evaluating a potential role of PIPAC as a neoadjuvant therapy in peritoneal metastasis patients not eligible for cytoreductive surgery and hyperthermic intraperitoneal chemotherapy (HIPEC). Although these pilot data should be considered preliminary and interpreted with caution, a few interesting observations can be done.

First, in our institution, PIPAC was performed much more frequently than CRS and HIPEC. In fact, there were 12 times more PIPAC than HIPEC procedures. This suggests that the indications for PIPAC might be more common than those for CRS and HIPEC. It has to be noted that, as a priority, we performed CRS and HIPEC in all eligible patients, rather than PIPAC. Thus, it appears that PIPAC can be proposed to many patients who are not eligible for CRS and HIPEC.

Second, we know from previous studies that about three quarters of patients with peritoneal metastasis in the salvage situation develop major or complete intraperitoneal tumor regression after repeated PIPAC therapy, as assessed by histology [10–13]. In the present cohort of patients, many patients were not primarily eligible for CRS and HIPEC because of the extent of peritoneal disease, in particular because of diffuse small bowel involvement. An important finding of this case series is that CRS and HIPEC were possible in a subgroup of patients after repeated PIPAC. Secondary CRS and HIPEC were indeed only possible in a small number of patients (5.1 % of all patients treated with PIPAC during the period of time under observation). However, this might be a message of hope for patients and HIPEC surgeons, showing that PIPAC might be able to control in some cases diffuse small bowel involvement, a feature considered as a critical to allow complete cytoreduction [27, 34]. Interestingly, the majority of these patients have peritoneal metastasis of colorectal cancer origin, suggesting a potential role of PIPAC as a neoadjuvant therapy in this particular indication.

Third, probability of overall survival was lower in the colorectal cancer patients having received neoadjuvant therapy with PIPAC before CRS and HIPEC. This observation is all but a surprise since these patients had a more extensive disease than patients who were treated primarily with CRS and HIPEC. In fact, the difference between both survival curves did not achieve significance. Although the number of patients is limited, this suggests that PIPAC might have a positive impact on survival in these selected patients. This hypothesis has now to be confirmed by adequate prospective studies.

Fourth, the mean number of PIPAC cycles needed to transform diffuse intraperitoneal metastasis into localized diseased approached four, or a time period of approximately 4.5 to 6 months. Clearly, peritoneal metastasis needs a relatively long time to go into regression under PIPAC therapy.

Finally, it has to be mentioned that three patients with signet-ring cell cancer (two with gastric cancer, one with appendiceal cancer) developed early and explosive tumor recurrence after secondary CRS and HIPEC, suggesting that this aggressive surgical procedure might not have been helpful in these patients. Although objective tumor control had been achieved by repeated PIPAC application, biology of signet-ring cell remains aggressive [35]. Creation of peritoneal wounds, stimulation of tumor growth, angiogenesis, and postoperative impairment of immune defenses might explain this observation. Clearly, further experimental and clinical work is needed on this question.

Taken together, these preliminary results suggest that PIPAC might be used as a neoadjuvant therapy before CRS and HIPEC in the future. Of course, the role of PIPAC and systemic neoadjuvant chemotherapy in peritoneal metastasis has to be evaluated in adequate, specific clinical trials. The aim of such neoadjuvant therapy would be to improve outcome of CRS and HIPEC, to select patients with chemosensitive, biologically favorable tumors, to extent the indications of CRS and HIPEC to some patients with diffuse small bowel involvement, and finally, to reduce the extent of surgery.

Against this framework, it has to be emphasized that the technical feasibility and the efficacy of PIPAC are largely depending on the degree of enteroenteral and enteroparietal adhesions. In 12 % of our patients, abdominal access was not possible due to adhesions. Moreover, only exposed peritoneal surfaces that can be reached by the aerosol can be treated with PIPAC. Although we are not able at this point of time to stratify response after PIPAC depending on the degree of adhesions, it might make sense to apply PIPAC as early as possible in the therapeutic chain. In our experience, it is possible to perform PIPAC before CRS and HIPEC in the vast majority of patients. The opposite is not true since massive adhesions develop usually after extensive peritonectomy. This is unfortunate for patients developing intraabdominal recurrence after incomplete cytoreduction during CRS and HIPEC.

Finally, it appears that PIPAC is able to stabilize quality of life of a significant number of patients with peritoneal metastasis [15]. This appears to be an important feature in the palliative setting. Against this framework, it has to be noted that a recent phase-2 trial [36] with PIPAC with low-dose cisplatin and doxorubicin combined with systemic palliative chemotherapy (XELOX) has shown a median overall survival (13 months) superior to the figures reported after CRS and HIPEC (7.9 months) [37], and this in unselected patients with a poorer risk profile. Thus, it is reasonable to formulate the hypothesis that some patients could have achieved the same survival with a better quality of life, if they were kept on PIPAC treatment instead of CRS and HIPEC.

## Conclusions

In conclusion, we propose that neoadjuvant PIPAC, combined or not with systemic chemotherapy, might be a promising approach for patients with peritoneal metastasis, and might allow to reduce diffuse peritoneal spreading to a more localized tumor involvement, so that secondary CRS and HIPEC might become possible. PIPAC is a simple, safe procedure and the tolerance of the procedure is usually excellent. Since PIPAC is repeated at 6-week interval, it creates proper methodological preconditions for assessing tumor response to locoregional and/or systemic chemotherapy. We will now design a clinical trial evaluating a potential place of PIPAC as a neoadjuvant therapy in gastric cancer with synchronous peritoneal metastasis.

## Abbreviations

CRS: Cytoreductive surgery
HIPEC: Hyperthermic intraperitoneal chemotherapy
PIPAC: Pressurized intraperitoneal aerosol chemotherapy
TRG: Tumor regression grading
